# Conducting Community-Based Qualitative Research With LGBTQ Older Adults of Color During COVID-19

**DOI:** 10.1093/geroni/igab046.2244

**Published:** 2021-12-17

**Authors:** Austin Oswald, Aundaray Guess

**Affiliations:** 1 CUNY Graduate Center, New York, New York, United States; 2 GRIOT Circle, Brooklyn, New York, United States

## Abstract

COVID-19 continues to transform the way scientists conduct research with study participants, particularly older adults who are at high risk of becoming seriously ill from the virus. For older adults who may be negatively affected by the digital divide, inclusive data collection practices become even more nuanced. Qualitative researchers moving their research into digital spaces must think critically about their use of technology, and how it affects the quality of data as well as the participant experience. This presentation highlights ethical and methodological considerations from a completely digital, community-based, qualitative research study conducted alongside LGBTQ older adults of color during COVID-19. Strategies to build and strengthen community partnerships are discussed along with challenges and opportunities for collecting data in the current digital landscape. Publicly available records are identified as a potential data source to understand the lives of LGBTQ older adults of color when in-person research is not feasible.

